# An update on the “empirical turn” in bioethics: analysis of empirical research in nine bioethics journals

**DOI:** 10.1186/s12910-018-0246-9

**Published:** 2018-02-07

**Authors:** Tenzin Wangmo, Sirin Hauri, Eloise Gennet, Evelyn Anane-Sarpong, Veerle Provoost, Bernice S. Elger

**Affiliations:** 1 Institute for Biomedical Ethics, University of Basel, Basel, Switzerland; 2 Faculty of Medicine, University of Basel, Basel, Switzerland; 3 Bioethics Institute Ghent, University of Ghent, Ghent, Belgium

**Keywords:** Bioethics, Empirical research, Literature review, Qualitative research, Quantitative research

## Abstract

**Background:**

A review of literature published a decade ago noted a significant increase in empirical papers across nine bioethics journals. This study provides an update on the presence of empirical papers in the same nine journals. It first evaluates whether the empirical trend is continuing as noted in the previous study, and second, how it is changing, that is, what are the characteristics of the empirical works published in these nine bioethics journals.

**Method:**

A review of the same nine journals (Bioethics; Journal of Medical Ethics; Journal of Clinical Ethics; Nursing Ethics; Cambridge Quarterly of Healthcare Ethics; Hastings Center Report; Theoretical Medicine and Bioethics; Christian Bioethics; and Kennedy Institute of Ethics Journal) was conducted for a 12-year period from 2004 to 2015. Data obtained was analysed descriptively and using a non-parametric Chi-square test.

**Results:**

Of the total number of original papers (*N* = 5567) published in the nine bioethics journals, 18.1% (*n* = 1007) collected and analysed empirical data. Journal of Medical Ethics and Nursing Ethics led the empirical publications, accounting for 89.4% of all empirical papers. The former published significantly more quantitative papers than qualitative, whereas the latter published more qualitative papers. Our analysis reveals no significant difference (χ2 = 2.857; *p* = 0.091) between the proportion of empirical papers published in 2004–2009 and 2010–2015. However, the increasing empirical trend has continued in these journals with the proportion of empirical papers increasing from 14.9% in 2004 to 17.8% in 2015.

**Conclusions:**

This study presents the current state of affairs regarding empirical research published nine bioethics journals. In the quarter century of data that is available about the nine bioethics journals studied in two reviews, the proportion of empirical publications continues to increase, signifying a trend towards empirical research in bioethics. The growing volume is mainly attributable to two journals: Journal of Medical Ethics and Nursing Ethics. This descriptive study further maps the still developing field of empirical research in bioethics. Additional studies are needed to completely map the nature and extent of empirical research in bioethics to inform the ongoing debate about the value of empirical research for bioethics.

## Background

Bioethics has come a long way from being a theoretical domain of philosophy to engaging in research questions that require empirical fieldwork. In “The Birth of the Empirical Turn in Bioethics”, Borry and colleagues [1] put forth three main reasons for the growing focus on empirical research in bioethics: dissatisfaction with the abstract philosophical nature of bioethics, increasing engagement with clinical ethics, and the growing prominence of evidence-based medicine in the 1990s. The role of empirical research for bioethics resides in its potential to inform abstract principles into workable practices and its capacity to ensure that bioethicists are in touch with the actual experiences of those affected [2–4].

In the last decade, a few publications have sought to present evidence that bioethics is becoming increasingly empirical, resulting in the use of the term ‘empirical turn in bioethics’ [5, 6]. Sugarman, Faden, and Boyce’s [7] seminal study laid the foundation of empirical research in this field. Their PubMed search for the period ranging from 1980 to 2005 concluded that 13% of all ethics publications in PubMed were related to empirical biomedical ethics. They defined such works as the application of social science research methods to the direct examination of issues of biomedical ethics (p. 21). Their study reported that empirical biomedical ethics publications had increased from 8% in the period of 1980–1984 to 16% in 2000–2005; and during this 25-year period, 11,776 published papers were deemed empirical. The limitation of this study was that the results were based on outputs obtained from the use of specific MESH terms and manuscripts were not individually evaluated.

A second and (probably) the key study evaluating the extent of empirical research in bioethics, in which each empirical paper was assessed, was that of Borry and colleagues [8]. Unlike the previous study, they explored publications in nine bioethics journals from 1990 to 2003. They found that 435 (10.8%) of the 4029 articles used empirical design, meaning that empirical data was collected and analyzed. The authors also noted an increase in the percentage of empirical papers published from 5.4% in 1990 to 15.4% in 2003; and a highly significant increase in empirical publications in the period 1997–2003 compared with 1990–1996.

DeBois and colleagues [9] also undertook a systematic review of the literature to capture research ethics data published in three non-ethics health journals from 2005 to 2006. Their review found that ethics content remained hidden as only 26 (2.2%) of the published articles contained relevant content. They concluded that the invisibility of ethics content in these journals was due to the use of irrelevant keywords, lack of discussion surrounding ethical implications, and publishing in non-ethics journals. Extrapolating these findings, the authors reasoned that an estimated 433 ethics articles published each year in health journals could remain hidden to readers interested in bioethics, a claim substantiated by the volume of manuscripts found in the literature search by Sugarman and colleagues [7].

The growing publication of empirical works in bioethics and along with it the increasing use of empirical research methodology in bioethics has resulted in discussions about this use in light of bioethics’ normative orientation [10, 11]. These discussions also dealt with questions as how an integration of the two can occur and uncertainties as to which empirical method should be used and how its rigor can be guaranteed [6, 12–14]. That is, scholars have highlighted the challenges that researchers may face when delving into empirical research and combining empirical data with normative approaches, or even drawing ethical conclusions based on obtained data [6, 10, 11]. At the same time, there are efforts to study how the normative and the empirical can and should coalesce to answer research questions of ethical significance [4, 15–19]. This integration of the empirical and normative is often termed “empirical ethics” or “empirical bioethics” [12]. Although several different means of integration has been proposed [12, 15, 16, 19, 20] and a recent review [21] documented the many ways in which “empirical ethics” is done, there is no consensus as to how this integration ought to manifest.

In this study, we depart from the normative-empirical discussions and instead seek to provide an update on the so-called ‘empirical turn in bioethics’ found in the study carried out by Borry and colleagues [8], using the same nine bioethics journals. This is important because no other study has to date sought to re-capture or further substantiate the data reported a decade ago. Such a substantiation is valuable to provide an insight into whether there *is* continued increase in empirical work in (at least) the same nine journals, if so, what *are* the characteristics of these empirical publications, for example, which methods are used. Therefore, our review begins where Borry and colleagues’ ended and continues through 2015. We aim to provide an update first, about whether this ‘empirical turn’ is continuing within the same nine journals and compare our review with that of our predecessor. Second, we intend to capture how this literature is changing, that is, what methods are being used in the empirical publications, their topics and the research subjects that are studied.

## Methods

### Data source and selection process

A review of the literature was undertaken using the following nine journals: Bioethics; Journal of Medical Ethics; Journal of Clinical Ethics; Nursing Ethics; Cambridge Quarterly of Healthcare Ethics (hereafter “Cambridge Quarterly”); Hastings Center Report; Theoretical Medicine and Bioethics; Christian Bioethics; and Kennedy Institute of Ethics Journal. These nine journals were selected in the previous review because they are dedicated to studying bioethics issues in healthcare and medicine [8, 22, 23].

Our review process began by retrieving titles and abstracts of all publications in these journals spanning the 12-year period from 2004 to 2015. A total of 7235 publications were collected from the nine journals and compiled in EndNote7. The first author read all entries (title and abstract where available). After exclusion of introductions, notes, comments, replies, and editorials (*n* = 1668), 5567 manuscripts formed the number of original works published. The inclusion criteria were kept the same as the previous review [8] to decide if an original work is empirical or not. The following eligibility criteria were applied: (a) collection of either qualitative and/or quantitative data; and (b) analysis of qualitative and/or quantitative data. By qualitative analysis we mean analytic methods used for open-ended non-numeric data such as content analysis, thematic analysis, and other qualitative techniques. Quantitative analysis refers to the use of statistical analysis for closed ended numeric data. A total of 4421 manuscripts did not meet our eligibility criteria for this study and were excluded from further analysis. The excluded documents included theoretical manuscripts without any empirical data collected and/or analysed, case studies describing information on a single patient, and literature reviews in which data from many studies were compiled. Although the latter two are, by strict definition, empirical, they were excluded to first maintain comparability with the previous review and second due to practical reasons associated with available resources to the researchers. The full-text articles of all the remaining manuscripts considered as empirical (*n* = 1146) were retrieved for analysis.

### Data extraction process

**Fig. 1:**
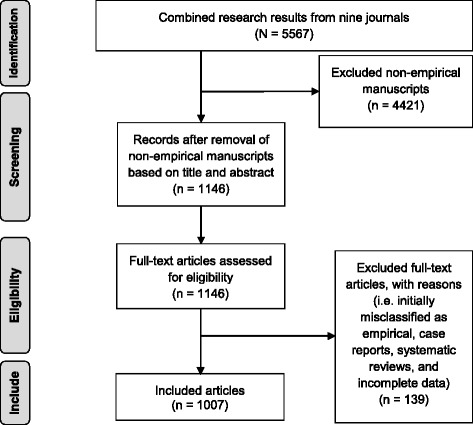
Search process for empirical manuscripts in the nine bioethics journals

Two authors examined the abstract and methods section of each of the 1146 manuscripts (Fig. 1). To standardize the quality of data captured from each publication, a data extraction sheet was developed. This form was used to collect the following information: title, number of authors, research area, country where the study was carried out, specific topic of research, participant characteristics, type of study (e.g. prospective or retrospective cross-sectional, longitudinal), type of research methodology (i.e. qualitative, quantitative, and mixed-methods), mode of data collection (e.g. survey, interviews, participant observation), participant sampling, number of participants, type of analytical method, persons engaged in the analysis, and if research participants received payment for their participation.

### Data entry, management, and analysis

The co-authors entered the retrieved data into IBM SPSS.22. The first author checked all entered data for accuracy against the data extraction sheet that she completed for each manuscript. Any manuscript deemed ineligible by two authors was also excluded. This resulted in the removal of 131 manuscripts (articles misclassified as empirical because the respective abstracts were not available during the identification stage, and case reports and literature reviews that we missed during that stage). A further eight manuscripts were removed for incomplete data, that is, the articles failed to provide clear methodological descriptions or no other material was available to discern the nature of the study; and for one manuscript, the full-text article could not be accessed. Thus, 1007 manuscripts comprised the full sample for data analysis. Data were first analysed descriptively (frequencies, mean, median, range; and cross-tabs) using IBM SPSS.22, then using a non-parametric Chi-square test to determine if there was an increasing empirical trend in bioethics. Statistical significance level was set a priori at *P* < .05.

## Results

### Empirical research in the nine bioethics journals: an increasing trend?

From the 1007 articles, 89.4% were published in two journals: Journal of Medical Ethics (*n* = 484; 48.1%) and Nursing Ethics (*n* = 416; 41.3%). Journal of Clinical Ethics ranked third with 5.5% (*n* = 55) of the total number of empirical research papers, followed by Bioethics (*n* = 25; 2.5%) and Cambridge Quarterly (*n* = 18; 1.8%). The remaining four journals contributed 3 or less empirical manuscripts (< 0.3%) in the 12 years of examination.

**Fig. 2:**
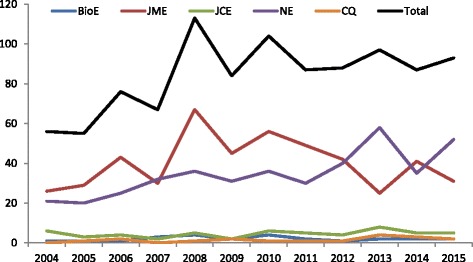
The number of empirical papers in the nine bioethics journals from 2004 to 2015* The following journals with negligible amount of empirical works are not represented in the figure: Theoretical Medicine and Bioethics, Christian Ethics, and Kennedy Institute of Ethics Journal. BioE = Bioethics; JME = Journal of Medical ethics; JCE = Journal of Clinical Ethics; NE = Nursing Ethics; HCR = Hastings Center Report; CQ = Cambridge Quarterly

By year of publication, empirical papers accounted for 14.9% of all publications in 2004 and slightly increased to 17.8% in 2015, peaking at 22.2% in 2008 (Fig. 2; Table 1). The overall prevalence of empirical manuscripts in the nine bioethics journals was 18.1%. More than half of all publications (57.3%) in Nursing Ethics were empirical in nature (with up to 74.3% in 2015), while 25.7% of published papers in the Journal of Medical Ethics and 14.4% in the Journal of Clinical Ethics were empirical (Table 1).

**Table 1:** Number (and proportion) of empirical manuscripts published in each of the nine bioethics journals between 2004 and 2015

Journal/ Year	JME n (%)	NE n (%)	JCE n (%)	BioE n(%)	CQ n (%)	KIB n (%)	HCR n (%)	TMB n (%)	CE n (%)	Total (%)**
2004	26 (23.2)	21 (43.8)	6 (13.6)	1 (4.0)	0 (−)	1 (4.5)	0 (−)	1 (5.6)	0 (−)	56 (14.9)
2005	29 (19.5)	20 (44.4)	3 (7.3)	1 (2.9)	1 (2.3)	0 (−)	0 (−)	1 (4.3)	0 (−)	55 (12.4)
2006	43 (29.1)	25 (56.8)	4 (11.4)	1 (4.5)	2 (5.6)	1 (5.6)	0 (−)	0 (−)	0 (−)	76 (18.5)
2007	30 (20.3)	32 (54.2)	2 (11.8)	3 (6.0)	0 (0.0)	0 (−)	0 (−)	0 (−)	0 (−)	67 (15.4)
2008	67 (31.6)	36 (60.0)	5 (23.8)	4 (7.3)	1 (2.6)	0 (−)	0 (−)	0 (−)	0 (−)	113 (22.2)
2009	45 (29.6)	31 (55.4)	2 (6.1)	2 (3.6)	2 (5.0)	1 (5.3)	1 (1.6)	0 (−)	0 (−)	84 (18.3)
2010	56 (33.1)	36 (59.0)	6 (18.8)	4 (6.7)	1 (1.7)	0 (−)	1 (1.6)	0 (−)	0 (−)	104 (20.9)
2011	49 (33.3)	30 (44.8)	5 (18.5)	2 (3.4)	1 (2.7)	0 (−)	0 (−)	0 (−)	0 (−)	87 (19.2)
2012	42 (28.2)	40 (57.1)	4 (15.4)	1 (1.7)	1 (3.4)	0 (−)	0 (−)	0 (−)	0 (−)	88 (19.7)
2013	25 (17.2)	58 (65.9)	8 (20.5)	2 (3.3)	4 (10.3)	0 (−)	0 (−)	0 (−)	0 (−)	97 (19.9)
2014	41 (24.3)	35 (60.3)	5 (17.9)	2 (3.7)	3 (5.4)	0 (−)	1 (1.0)	0 (−)	0 (−)	87 (16.4)
2015	31 (16.8)	52 (74.3)	5 (12.5)	2 (2.7)	2 (6.3)	0 (−)	0 (−)	0 (−)	1 (4.5)	93 (17.8)
Total (%)*	484 (25.7)	416 (57.3)	55 (14.4)	25 (4.1)	18 (3.6)	3(1.4)	3 (0.4)	2(0.7)	1 (0.5)	1007 (18.1)

*JME* journal of medical ethics, *NE* nursing ethics, *JCE* journal of clinical ethics, *BioE* bioethics, *NE* nursing ethics, *CQ* Cambridge Quarterly, *KIB* Kennedy Institute of Ethics Journal, *HCR* hastings center report, *TMB* theoretical medicine & bioethics, *CE* Christian Bioethics

^*^Empirical papers/Total original works published in the journal

**Empirical paper/total original papers published by year

Similar to the previous review [8], we tested if there was an increase in the number of empirical publications during the search period by creating a dichotomous, categorical time variable: (a) 2004–2009 and (b) 2010–2015. The findings of our non-parametric chi-square test for the entire dataset (*N* = 5567; empirical *n* = 1007; non-empirical 4560) reveal no significant difference (χ2 = 2.857; *p* = 0.091) between the number of empirical papers published in 2004–2009 (*n* = 451) and 2010–2015 (*n* = 556).

**Fig. 3:**
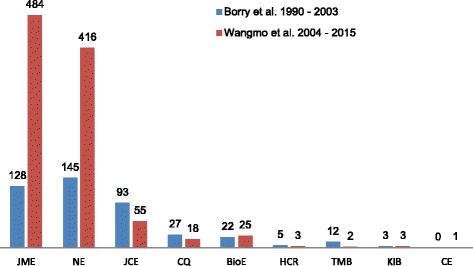
Comparing empirical papers published in the nine bioethics journals in the two time periods* JME = Journal of Medical ethics; NE = Nursing Ethics; JCE = Journal of Clinical Ethics; BioE = Bioethics; NE = Nursing Ethics; CQ = Cambridge Quarterly; KIB = Kennedy Institute of Ethics Journal; HCR = Hastings Center Report; TMB = Theoretical Medicine & Bioethics; CE = Christian Bioethics

Figure 3 shows our results, by journal, also in comparison with the previous review [8]. It highlights that Journal of Medical Ethics and Nursing Ethics have accepted more empirical manuscripts in our search period of 2004–2015 than the previous review’s search period of 1990–2003. The data presented in Table 1 and Fig. 3 also underline that the remaining seven journals have continued to publish very low to negligible proportions of empirical research papers, and thus remain predominantly oriented toward normative ethics publications.

### The nature of empirical research: Type of method and choice of journals

The methods applied in the papers were categorized into 3 groups: qualitative, quantitative, and mixed methods (Table 2). Qualitative studies were those that gathered non-generalizable data using methods such as face-to-face, in-depth, unstructured or semi-structured interviews; focus group discussions; and participant observations, or those that carried out qualitative analysis using written policies and guidelines or from open-ended survey questions. Quantitative studies included close-ended surveys (interviewer administered or self-completed) and experimental designs. Of the 1007 manuscripts, 427 studies (42.4%) were classified as using qualitative methods, and 533 published works (52.9%) were classified as using quantitative methods. Mixed methods studies were those which used both qualitative and quantitative methods. Only 47 studies (4.7%) were categorized as such. Studies that obtained data using one method but carried out both qualitative and quantitative analysis were not categorized as mixed methods. Most qualitative papers were published in Nursing Ethics (Table 2), which published significantly more qualitative than quantitative papers (χ2 = 8.724; *p* = .003). Inversely, the Journal of Medical Ethics published twice as many quantitative as qualitative papers (χ2 = 59.056; *p* = .000) and accounted for most of the quantitative papers published.

**Table 2:** Type of methods used per paper published in the nine journals

Methods / Journals	Quantitative	Qualitative	Mixed methods	Total
JME	313	148	23	484
NE	170	229	17	416
JCE	27	23	5	55
BioE	11	14	0	25
CQ	9	8	1	18
KIB	1	2	0	3
HCR	1	1	1	3
TMB	0	2	0	2
CE	1	0	0	1
Total	533	427	47	1007
%	52.9%	42.4%	4.7%	100%

*JME* journal of medical ethics, *NE* nursing ethics, *JCE* journal of clinical ethics, *BioE* bioethics, *NE* nursing ethics, *CQ* Cambridge Quarterly, *KIB* Kennedy Institute of Ethics Journal, *HCR* hastings center report, *TMB* theoretical medicine & bioethics, *CE* Christian Bioethics

Surveys (*n* = 468; 46.5%) were the most used mode of data collection followed by qualitative face-to-face interviews (*n* = 285; 28.3%). Little over one-tenth of the study (*n* = 117; 11.6%) used more than one tool to collect data, e.g., interviews and focus groups. The remaining mode of data collection were documents (*n* = 83; 8.2%) which mostly included files from research ethics committees, medical records, protocols, and guidelines; focus groups (*n* = 50; 5.0%); and participant observation (n = 4; 0.4%).

### The where, who, and what of empirical research in bioethics

For close to half of the 1007 total papers, the data was collected in Western Europe (47.6%), followed by North America (21.8%), Middle-East (8.6%), Asia (8.5%), Australia and New Zealand (3.6%), Africa (2.3%), Eastern Europe (2.3%), and South America (1.6%). For 3.7% of the studies, data was collected from more than one country within one of the above eight categories. The ten most represented countries from which the study participants hailed were USA (*n* = 171); UK (*n* = 108); Sweden (n = 83); Norway (*n* = 65), the Netherlands (*n* = 62), Canada (*n* = 41), Turkey (*n* = 37), Finland (*n* = 36); Iran (*n* = 28), and Australia (n = 28).

Health care professionals (i.e. nurses and doctors) were the most studied population (36.6%), followed by patients (13%), and students (8.4%). Other groups included general population (5.9%), family members (4.2%), ethics committee members (3.3%), and children (0.9%). We also included an “Other or combination” category (27.4%), which denotes that the study either gathered information on more than one participant group or was a document analysis for which no participants were specified.

Concerning authorship, 100 empirical manuscripts (9.9%) were by a single author, 182 (18.1%) by two authors, 244 (24.2%) three authors, 202 (20.1%) four authors, and the remaining 27.7% were authored by five or more persons. The mean number of authors was 3.7 (S.D. 2.0; Range: 1–18). By type of methodology, 246 (60.3%) qualitative papers had 3 authors or less and similarly, 242 (45.5%) quantitative papers had 3 authors and less. For those papers that had 4 authors or more, 162 (39.7%) used qualitative design whereas 290 (55.5%) were quantitative in nature.

More than 80 different topics were explored in these empirical papers. We have collated them into 25 main topics (see Table 3) using categorization evident in the previous publication [8] as well as that of the Bioethics Research Library Category Scheme, Georgetown University.

**Table 3:** Topics examined in empirical manuscripts published in the nine bioethics journals

Main topic	Frequency
Informed consent (including information provision and participation)	106
Palliative care, Euthanasia, Assisted Suicide	99
Theoretical perspectives on ethical behaviors	75
Medical communication and decision making	72
Ethics education and training	67
Professional ethics	67
Research ethics	60
Care ethics	52
Caring for vulnerable groups	49
End of life decision making	47
Genetic research and testing	34
Relationships (doctor-patient/Professional-professional)	33
Healthcare organization and resource allocation	32
Confidentiality and privacy	30
Dignity and Autonomy	30
Public health ethics	23
Organizational climate	23
Organ donation and transplantation	20
Religion and culture	20
Death/Suicide/Autopsy	14
Reproductive ethics	14
Involuntary care	12
Ethics of various broad disciplines	9
Quality of life	4
Other topics	15
TOTAL	1007

## Discussion

Our results put forth the following four valuable findings for scholars working in the field of empirical research in bioethics and “empirical bioethics”: (a) an increase in the absolute number of empirical studies published in the nine journals; (b) a slower rate of growth of empirical research published in the nine journals; (c) the dominance of quantitative studies; and (d) the attention for experiences of the studied sample, that is, health care providers and patients. It thus presents much needed insight into where the field is going. It also provides the necessary data to inform discussions about the value and the methods of empirical research in bioethics.

First, although the difference in the proportion of empirical papers published between two time periods (2004–2009 vs. 2010–2015) was insignificant (*p* = 0.091), the proportion of empirical research publications increased from 14.9% in 2004 to 17.9% in 2015. In absolute terms, the numbers of empirical papers that we found (*N* = 1007) has more than doubled when compared to the previous review (*N* = 435). This higher volume could be attributed to the generally greater number of empirical papers accepted in these two journals but it also suggests that, in absolute numbers, more empirical research is being carried out in the field. In light of the latter, it is time that we address the question concerning the appropriateness of the empirical methods used and the quality of the data collections and analyses. This type of scrutiny has not been done (to our knowledge) and is needed to shed light on the methodological rigor issue that have been raised by other scholars [6, 14, 24].

Comparing our findings with the previous study, we also find that the increased volume of empirical research in bioethics in our search is mainly attributable to two journals: Journal of Medical Ethics and Nursing Ethics. The proportion of empirical articles in Nursing Ethics has increased from 39% between 1990 and 2003 [8] to 57.3% between 2004 and 2015. The same is true for the Journal of Medical Ethics, for which, the proportion of empirical publications has risen from 16.8% in 1990–2003 to 25.7% in 2004–2015.

Second, our findings suggest that although there is a trend toward more empirical research in these nine bioethics journals, the rate of growth is slowing. Specifically, for the quarter century data that is now available from the search of these nine journals from the two separate reviews, we know that 10.9% used an empirical design in the previous review [8], and 18.1% in this current review, with a peak in 2008 at 22%. However, we find a slower growth of empirical publications, that is, 14.9% in 2004 to 17.9% in 2015 in our search, when compared to the three-fold increase from 5.4% in 1990 to 15.4% in 2003 observed in the previous search [8]. Sugarman and colleagues’ search [7] noted that, in their 25 years of examination, 13% of the publications about ethics contained empirical research. They also reported that the proportion of empirical research in bioethics might actually be slowing after reaching its peak at 21% (see p. 26 [7]).

When compared to the previous review [8], for seven journals (excluding Journal of Medical Ethics and Nursing Ethics) the uptake of empirical articles has remained minimal. That is, these journals have accepted empirical research in bioethics at a very low (i.e. Bioethics, Journal of Clinical Ethics; Cambridge Quarterly) or negligible rate (i.e. Theoretical Medicine and Bioethics; Hastings Center Report; Christian Ethics; and Kennedy Institute of Ethics Journal). This may also be a reason for the ‘slower’ trend that we found. However, it may also be highlighting the fact that empirical research in bioethics is not considered (by Editorial boards or researchers) as falling within the scope of these seven journals and that in the last decade empirical researchers in bioethics have sought to simply publish their empirical manuscripts elsewhere.

Third, concerning the empirical methods used, studies with quantitative methods remained predominant in the nine bioethics journals. However, their dominance has decreased from 65% of all empirical papers in the previous search [8] to 53% in our search, whereas the amount of qualitative research publications have increased from 32.2% to 41% of all empirical papers, respectively. There were more quantitative papers with 4+ authors than qualitative papers with 4+ authors, a finding that has been corroborated by others [23]. In our study, close-ended surveys are the most used form of data collection. This was true for the other two studies [7, 8] as well, followed by face-to-face interviews. As was noted by our predecessors [8], the Journal of Medical Ethics’ affinity towards quantitative methods and Nursing Ethics’ towards qualitative methods might be explained by their disciplinary heritage. Nevertheless (as stated above) questions must be raised concerning the quality of methods used. This is especially significant because bioethicists borrow methods from the social sciences and many researchers working in the field of bioethics may not have been trained in empirical research methodology [25]. At the same time, the field must also evaluate whether there are methods (particular qualitative or quantitative) that are more suitable to carry out a normative-empirical integration, thereby forwarding the debates related to when and how to do “empirical bioethics” [12, 26].

Forth and finally, the empirical research published in the nine bioethics journals studied the experiences of physicians, nurses, and patients, who constituted approximately half of the study sample. This pattern in study sampling highlights the interests in and thereby significance attached to their knowledge and experiences. Also, the use of more than one participant group in more than a quarter of the studies may be underlining the significance placed on studying the same topic from the perspective of different stakeholders. Such developments are indeed positive as they aim to ensure the relevance of bioethics to the field of medicine, a core reason for the turn toward empirical research [1, 3, 12].

### Limitations

We caution our readers to refrain from generalization since the current state of the art available from the two reviews is based on evaluation of nine journals only. There are bioethics journals in other languages (e.g. Ethik in der Medizin), bioethics works published in books or book chapters that we did not evaluate. Additionally, there are 41 English language journals in bioethics (for list see https://bioethics.georgetown.edu/using-the-library/bioethics-journals/). In fact, our work represents only 22% of the English language bioethics journals. To make this caution clearer, we ran a preliminary scan of published papers in BMC Medical Ethics and Journal of Bioethical Inquiry for the 3 year period from 2014 to 2016 and found that 60% (153/254) of the publications in the former have used empirical methods, while empirical papers accounted for only 14% (23/169) of the latter. BMC Medical Ethics has 494 publications since its inception in 2000 and Journal of Bioethical Inquiry reports 811 publications^1^ since its inception in 2004.

As stated previously, the choice of limiting the review to the same journals as the first review on this topic [8] risks introduction of bias. This decision was made, however, to guarantee comparability between the two reviews and to provide an update on the increasing empirical research publication that was documented previously. Moreover, this focus allowed us to obtain data from nine bioethics journals which has resulted in an accumulation of data for a period of a quarter century (collected in two separate reviews). Apart from comparability, this choice to limit the review to the same nine journals was also made for reasons of feasibility. Inclusion of other (newer) bioethics journals would have increased the volume of empirical papers to a level where assessment by several researchers would require time and resources that were not available, potentially risking the rigor of the study.

### Future research

For further research, for instance, the inclusion of bioethics journals such as Developing World Bioethics and those with broader health focus, such as Public Health Ethics, might allow researchers to capture topics (e.g. concerns of other populations) that remained excluded in these nine journals. As is evident from our results, approximately 4/5 of the empirical data published in the nine bioethical journals were collected in Western Europe, USA, and Canada. As such, empirical publications in these journals were not truly international [22]. Second, it would also be interesting to elucidate if this “empirical turn” is present in nascent bioethics journals that were not captured in this study (e.g., Journal of Bioethical Inquiry, BMC Medical Ethics), legal ethics journals, and engineering and science ethics journals. Third, it might be crucial to explore empirical ethics publications in healthcare and medical journals whose core audience are not ethicists (a focus that is similar to the review done by DuBois and colleagues [9]). Such an investigation will provide valuable data on amount and scope of the empirical bioethics studies which are meant for non-ethics audience and thus not even captured in studies such as ours. Forth, we did not examine whether a normative analysis was carried out in the empirical studies that we found. This would be a praiseworthy endeavour for future research as it will add to the growing literature base on “empirical bioethics” [12, 18, 20, 21]. Finally, since the methods and their respective approaches (for qualitative methods: phenomenology, thematic analysis, content analysis, grounded theory) stem from other disciplines (e.g. sociology, anthropology, and psychology), it is important to examine whether the rigor and theoretical assumptions embedded within the methods used and their respective approaches have been upheld [6, 27, 28] by empirical research in bioethics.

## Conclusions

In the quarter century of data now available from the nine bioethics journals studied in two separate reviews, the amount of empirical publications continues to increase, albeit at a reduced pace, indicating a trend toward empirical research in bioethics. The findings that we have so far illustrate that the field is not and will possibly never be a solely normative field again. We also conclude that this trend noted so far from the nine journals captured is driven by two journals (Journal of Medical Ethics and Nursing Ethics). Hence, seven of the nine bioethics journals evaluated in the two reviews continue to be normatively oriented and publish much greater proportions of non-empirical manuscripts than empirical research in bioethics. Thus, to truly capture the scope and nature of ‘empirical turn in bioethics’, studies examining a wider range of journals, including new and emerging bioethics journals as well as empirical bioethics work in non-bioethics journals will be necessary. These studies would provide valuable information to further map the field of empirical work in bioethics and may result in future studies that delve into the debate about the methodological questions related to the rigor of empirical methods used, and when and how the integration of the normative and the empirical can be done.

## Footnotes

1 The search for both journals was completed on 03.04.2017. The total number includes editorials, correspondence, erratum, book reviews, and film reviews etc.
